# Anticancer potential of phytochemicals from *Oroxylum indicum* targeting Lactate Dehydrogenase A through bioinformatic approach

**DOI:** 10.1016/j.toxrep.2022.12.007

**Published:** 2022-12-14

**Authors:** Sheikh Sunzid Ahmed, M. Oliur Rahman, Ali S. Alqahtani, Nahid Sultana, Omer M. Almarfadi, M. Ajmal Ali, Joongku Lee

**Affiliations:** a Department of Botany, University of Dhaka, Dhaka 1000, Bangladesh; b Department of Botany, Jagannath University, Dhaka 1100, Bangladesh; c Department of Pharmacognosy, College of Pharmacy, King Saud University, P.O. Box 2457, Riyadh 11451, Saudi Arabia; d Deperment of Botany and Microbiology, College of Science, King Saud University, Riyadh 11451, Saudi Arabia; e Department of Environment and Forest Resources, Chungnam National University, Daehak-ro, Yuseong-gu, Daejeon, Republic of Korea

**Keywords:** Lactate Dehydrogenase A, Cancer, Molecular docking, Molecular dynamics simulation, MM/GBSA, *Oroxylum indicum*

## Abstract

In recent years, small molecule inhibition of LDHA (Lactate Dehydrogenase A) has evolved as an appealing option for anticancer therapy. LDHA catalyzes the interconversion of pyruvate and lactate in the glycolysis pathway to play a crucial role in aerobic glycolysis. Therefore, in the current investigation LDHA was targeted with bioactive phytochemicals of an ethnomedicinally important plant species *Oroxylum indicum* (L.) Kurz. A total of 52 phytochemicals were screened against LDHA protein through molecular docking, ADMET (Absorption, Distribution, Metabolism, Excretion and Toxicity) assay and molecular dynamics simulation to reveal three potential lead compounds such as Chrysin-7-O-glucuronide (−8.2 kcal/mol), Oroxindin (−8.1 kcal/mol) and Oroxin A (−8.0 kcal/mol). ADMET assay unveiled favorable pharmacokinetic, pharmacodynamic and toxicity properties for all the lead compounds. Molecular dynamics simulation exhibited significant conformational stability and compactness. MM/GBSA free binding energy calculations further corroborated the selection of top candidates where Oroxindin (−46.47 kcal/mol) was found to be better than Chrysin-7-O-glucuronide (−45.72 kcal/mol) and Oroxin A (−37.25 kcal/mol). Aldolase reductase and Xanthine dehydrogenase enzymes were found as potential drug targets and Esculin, the FDA approved drug was identified as structurally analogous to Oroxindin. These results could drive in establishing novel medications targeting LDHA to fight cancer.

## Highlights

• High throughput virtual screening protocol was followed for molecular docking of phytochemicals of *Oroxylum indicum*.
• ADMET assay revealed pharmacokinetic and pharmacodynamic properties of Chrysin-7-O-glucuronide, Oroxindin and Oroxin A.
• 100 ns molecular dynamics simulation unveiled structural stability and compactness.
• Prime MM/GBSA supported the selection of lead compounds with high free binding energies.

## Graphical Abstract

**Figure:**
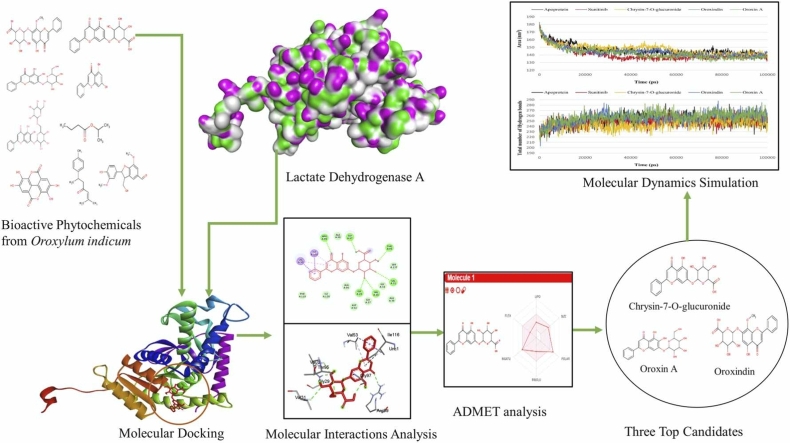

## 1 Introduction

Cancer is one of the major causes of mortality worldwide, accounting for approximately 10 million deaths in 2022, as reported by the WHO (https://who.int/news-room). Lactate Dehydrogenase A (LDHA) enzyme is considered as a key player in cancer progression and is targeted frequently to develop anticancer medications [1]. Cancer cells often switch their metabolic activities from oxidative phosphorylation to enhanced glycolysis [2]. Glycolysis is the breakdown of a whole glucose molecule into two pyruvate molecules. In cancer cells, glycolysis process breaks down glucose molecules partially and produces pyruvate molecules which are then transformed into lactate via a metabolic mechanism catalyzed by Lactate Dehydrogenase (LDH) enzyme [3]. This lactate synthesis is responsible for increased glycolysis, which contributes significantly to cancer growth and progression by lowering the pH for invasion, replenishing NAD+ for glycolysis, and inducing immune escape [4].

LDH structure is well preserved across species, with very minor alterations in amino acid sequences [5], and the structural affinity of LDH provides a justification for developing small-molecule inhibitors to modify its catalytic action in cells. LDH’s active pocket contains catalytically active residues such as His193, Asp168, Arg171, Thr246, and Arg106 [6]. LDH possesses five isozymes where the fifth form or LDH-5, also known as LDHA is upregulated in most of the tumor cells, thus inhibiting LDHA decreases tumor development and invasiveness [7]. LDHA expression has been dysregulated in endometrial cancer cells, squamous cell carcinoma and breast cancer cells [8], [9], [10], [11]. Abnormal activation of these oncogenes may result in increased glucose absorption and lactate generation. Inhibition of LDHA, therefore, may limit the energy supply in tumors, reducing cancer cells' ability to spread and invade. As a result, LDHA is gaining popularity as a possible diagnostic or prognostic biomarker for cancer, as well as a therapeutic target for the development of future anticancer medicines [12], [13], [14], [15], [16].

*Oroxylum indicum* (L.) Kurz is a small to medium tree belonging to the family Bignoniaceae, and is native to Bangladesh and Indian subcontinent. The tree can attain a height of up to 12 m. The species is characterized by its pinnately compound leaves, ovate leaflets, pinkish red flowers in terminal raceme inflorescence, axile placentation and boat-shaped, sword-like fruits [17], [18]. Traditional medicinal knowledge (TMK) has reported various components of this plant to cure different maladies including cancer [19]. Several *in vivo* and *in vitro* investigations have revealed its anticancer, antiulcer, hepatoprotective, anti-inflammatory, and immunostimulant properties [18]. *O. indicum* has been shown to possess a wide variety of bioactive phytochemicals, viz., alkaloids, flavonoids, cardiac glycosides, phenols, and other bioactive compounds that can help to accelerate the discovery of nature-based new LDHA inhibitors.

Computer-aided structure-based drug design approach has revolutionized the discovery of drugs by expediting the process through bioinformatic servers and tools involving molecular docking, ADME/T (Absorption, Distribution, Metabolism, Excretion and Toxicity) analyses and molecular dynamics simulation. This strategy is specific and effective in finding and refining new lead compounds and thus has contributed to enhance our current understanding of the discovery of novel therapeutics [20]. Molecular docking as a computational modeling approach elucidates the interactions between a small molecule (as ligand) and a protein (as receptor) at the atomic level, which permits to characterize the behavior of small molecules in the binding site of target protein as well as to understand fundamental biochemical processes [21]. Molecular docking relies on two basic steps – firstly, by sampling conformations of the ligand in the active site of the protein and secondly, ranking these conformations via a scoring function [22]. Scoring functions help to differentiate the correct poses from incorrect poses, or binders from inactive compounds in a reasonable computation time. Different methodologies are used for molecular docking such as, rigid ligand and rigid receptor docking, flexible ligand and rigid receptor docking, flexible ligand and flexible receptor docking and so on [23]. Molecular docking has been the most widely employed computational technique and the main application of the approach lies in the structure-based drug designing for identification of new active compounds towards a specific target protein [24]. ADME/T analyses is supportive to understand whether the pharmacokinetic (PK) and pharmacodynamic (PD) properties of the lead compounds are in the acceptable range or not. Pharmacokinetic properties examine how the drug is absorbed, distributed, metabolized, and excreted by the body, while pharmacodynamic properties interpret the relationship between drug concentration at the site of action and the resulting effect, including the time course and intensity of therapeutic and adverse effects [25]. A shocking 90% attrition rate of drug candidates is reported by the pharmaceutical industry during the transition from preclinical trials to phase 4 clinical trials [26]. The main causes of the high failure rate of drug discoveries include undesirable bioavailability of drugs due to inappropriate pharmacokinetic and pharmacodynamic properties. During the synthesis of drug molecules, a delicate balance between drug candidates and their ADME/T profiling can help prevent late-stage drug failure in the drug discovery process [27]. Therefore, earlier PK/PD property detection in conjunction with drug-likeness and ADME/T profiling can save time and money while simultaneously assuring the stability and safety of the designed drugs or candidate pharmaceuticals [28].

Though a few efforts on *in silico* have been made to explore medicinal properties of bioactive phytochemicals of *O. indicum*, no attempts have been taken so far to expose its anticancer efficacy targeting Lactate Dehydrogenase A. Therefore, the present study aimed at evaluating the bioactive compounds of *O. indicum* to unveil novel drug candidates targeting LDHA through bioinformatic approach.

## 2 Materials and methods

### 2.1 Target protein retrieval and preparation

Lactate Dehydrogenase A (LDHA) was retrieved from the RCSB (Research Collaboratory for Structural Bioinformatics) Protein Data Bank (PDB) database using the PDB ID '4OJN' (https://www.rcsb.org) [29]. The selected protein ‘4OJN’ was solved experimentally using X-ray diffraction method with eight chains. The resolution and observed R-value was 2.40 Å and 0.215, respectively [30]. BIOVIA Discovery Studio Visualizer v21.1.0.20298 was employed for removing all the chains from the protein except the chain A. Subsequently, all the heteroatoms including water molecules and two small molecules such as Pentaethylene glycol and Glycerol were removed. Afterwards, the receptor was modified using MGL-AutoDockTools v.1.5.6 and energy minimized using SWISS-PDB viewer v4.10 employing GROMOS96 43b1 force field. The PDB file from SWISS-PDB Viewer was converted to PDBQT format using Open Babel v.2.3.1 before performing docking [31], [32], [33], and the command has been provided in the Supplementary file 1.

### 2.2 Preparation of ligand library

Fifty-two phytochemicals of *O. indicum* were selected along with the control Sunitinib after thorough literature survey using SCOPUS, Google Scholar and PubMed databases to construct the ligand library for molecular docking and subsequent analyses [18], [19], [34]. All the phytochemicals and the control were retrieved from the PubChem database (https://pubchem.ncbi.nlm.nih.gov) in 3D SDF (Structure Data File) format. Afterwards, energy minimization was conducted for all the ligands employing MMFF94 force field in 2000 steps employing steepest descent algorithm of the Open Babel v.2.3.1 software in Linux Ubuntu 18.04.6 LTS environment using specific command (Supplementary file 1) [33], [35], [36]. Subsequently, all the ligands were converted from SDF to PDBQT format in the same Linux environment using necessary commands (Supplementary file 1).

### 2.3 Virtual screening through molecular docking

Molecular docking was carried out employing AutoDock Vina v.1.2.0 [37] in Linux command line along with the Perl programming script “Vina_windows.pl”, the script has been provided in the supplementary file 2. The receptor macromolecule was considered as rigid, whereas the ligands as flexible during blind docking. Grid box was constructed using MGL-AutoDockTools v.1.5.6 and the box covered the entire surface of the macromolecular receptor. Grid box was constructed before running the Perl script, and the size coordinates were 45.45 × 84.07 × 54.28, and the center coordinates were 70.33 × −15.09 × −26.45 for X, Y, and Z axes, respectively. Phytochemicals having similar or better scoring than Sunitinib were processed for further investigation. BIOVIA Discovery Studio Visualizer was used for visualization of the docked protein-ligand complexes.

### 2.4 Evaluation of Drug profile through ADME and toxicity analyses

After initial screening, 18 phytochemicals were selected for drug profile evaluation via ADME/T (Absorption, Distribution, Metabolism, Excretion and Toxicity) analysis. SwissADME server was employed to analyze various pharmacokinetic and pharmacodynamic properties related to ADME analysis for their vital role in determining the pharmacological activity and performance of drugs [38]. For accurate prediction, Canonical SMILES format files were produced for all the top 18 phytochemicals using Open Babel v.2.3.1 in the Linux command line, which were subsequently imported to the SwissADME server for predicting ADME properties. For toxicity analysis, two different servers were utilized, e.g. ProToxII and StopTox [39], [40]. ProTox-II server predicts various toxicity endpoints, such as acute toxicity, hepatotoxicity, cytotoxicity, carcinogenicity, mutagenicity, immunotoxicity, adverse outcomes (Tox21) pathways, and toxicity targets using fragment propensities, molecular similarity, most frequent features, and machine learning methods. StopTox server utilizes an ensemble of quantitative structure-activity relationship (QSAR) models to evaluate the toxicity of compounds for various toxicity end points including acute inhalation toxicity, acute oral toxicity, eye irritation and corrosion, skin sensitization etc. after compiling, curating and integrating the largest publicly available datasets.

### 2.5 Molecular dynamics simulation

Molecular dynamics (MD) simulation was performed for the top selected protein-ligand complexes for 100 ns using the GROMACS v2019.2 software via WebGro server (https://simlab.uams.edu) [41]. Before performing simulation, initially ligand topology files were generated using PRODRG server [42]. SPC (Simple Point Charge) water model was used to solve the system with triclinic box. The system was neutralized by sufficient sodium and chloride ions (0.15 M salt). GROMOS96 43a1 force field in 5000 steps was used for energy minimization [43]. For equilibration and MD run, NVT/NPT was used fixing the temperature at 300 K and pressure at 1 bar. V-rescale, a modified Berendsen thermostat was utilized for temperature coupling. Leap-frog method, as MD integrator, was employed for updating positions and velocities [44]. Finally, a 100 ns MD production run was performed setting approximate number of frames 1000 per simulation. MD simulation results were retrieved from the server in CSV format and plotted subsequently using Microsoft Excel V.2013 to analyze RMSD (Root Mean Square Deviation), RMSF (Root Mean Square Fluctuation), Rg (Radius of Gyration), SASA (Solvent Accessible Surface Area) (total) and number of hydrogen bonds between the LDHA and lead compounds.

### 2.6 Molecular mechanics/generalized born surface area (MM/GBSA)

Prime module of Schrödinger v.2021–2 software package was used to calculate free binding energies of the top selected complexes [45]. The Prime module employing OPLS2005 force field [46] and VSGB continuum solvation model was applied to calculate free binding energies using the following formula:

ΔG(bind) = ΔG(solv) + ΔE(MM) + ΔG(SA)

where ΔG(solv) indicates the difference in GBSA solvation energy of the protein-inhibitor complex and the sum of the solvation energies for unliganded protein and inhibitor; ΔE(MM) indicates difference in the minimized energies between protein-inhibitor complex and the sum of the energies of the unliganded protein and inhibitor; and ΔG(SA) indicates the difference in surface area energies of the complex and the sum of the surface area energies for the unliganded protein and inhibitor.

### 2.7 Drug target class and structurally similar analogs prediction

**Fig. 1:**
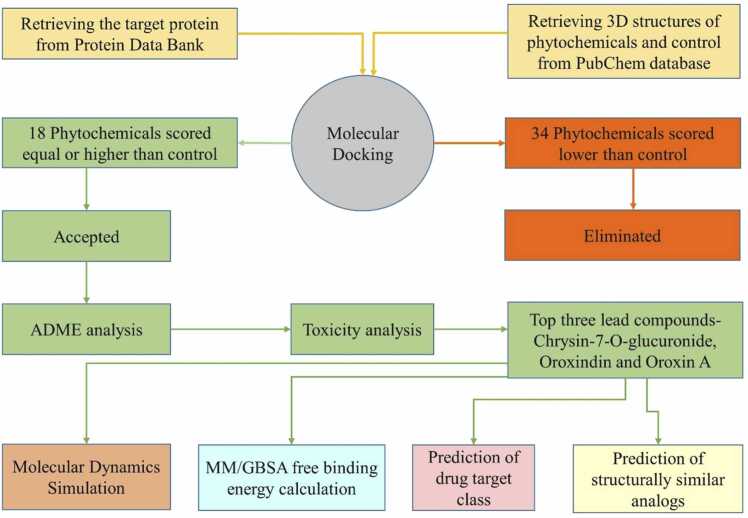
Workflow of the current study showing stepwise *in silico* screening of bioactive phytochemicals of *O. indicum* targeting Lactate Dehydrogenase A (LDHA).

SwissTargetPrediction server was utilized to predict potential macromolecular targets for the top selected candidates [47]. The server utilizes a library of 3,76,342 identified bioactive chemicals on roughly 3068 proteins on the basis of 2D and 3D similarity. The SwissSimilarity server was used to identify potential structural analogs that may be repurposed against the chosen receptor [48]. We employed the pathbased FP2 fingerprinting method on the DrugBank compound library (https://go.drugbank.com) to uncover bioactive compounds that might be used against the specified receptor. The SwissSimilarity service contains a screenable library of 2108 virtual compounds and applies a variety of complimentary methodologies such as chemical fingerprinting, superpositional 3D shape similarity, and rapid non-superpositional 3D form similarity. The workflow of present investigation has been elucidated in Fig. 1.

## 3 Results

### 3.1 Screening of phytochemicals via molecular docking

**Fig. 2:**
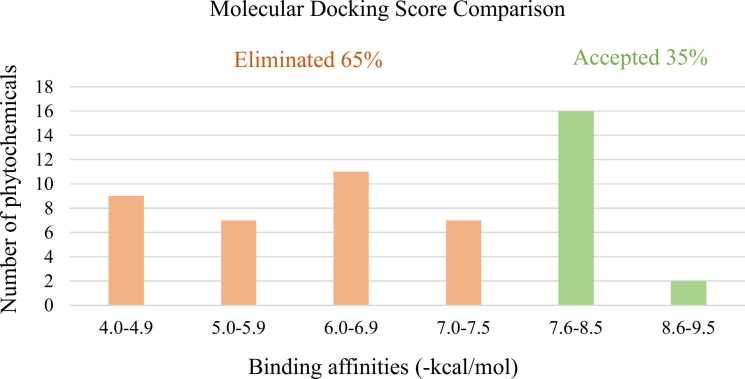
Binding affinities of the investigated phytochemicals via virtual screening.

**Fig. 3:**
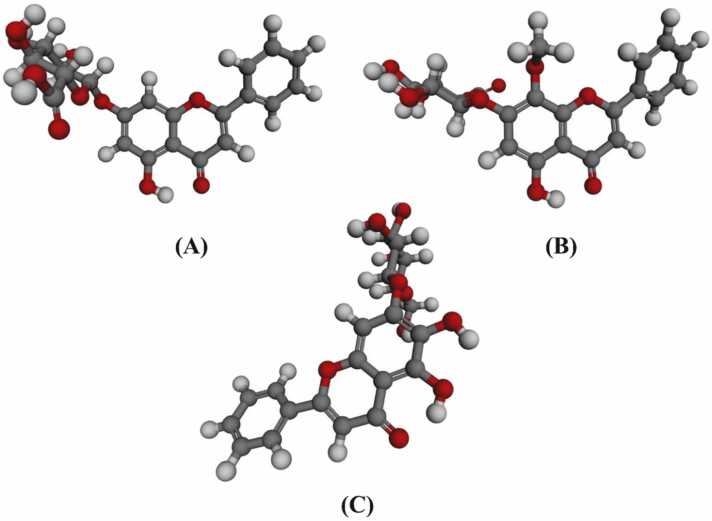
Three dimensional structures of the top selected lead compounds: (A). Chrysin-7-O-glucuronide; (B). Oroxindin and (C). Oroxin A.

The present study revealed that all the 52 phytochemicals were docked successfully with binding affinities ranging from − 4.0 to − 9.3 kcal/mol. The control Sunitinib scored − 7.6 kcal/mol when docked with LDHA (Table 1). A total 18 phytochemicals (35%) were accepted for scoring equal or higher than the control Sunitinib, while 34 phytochemicals (65%) were eliminated due to their low scores as compared to the control. Among the accepted phytochemicals, the highest binding affinity was found in Oroxin B (−9.3 kcal/mol). The remaining 16 accepted phytochemicals showed variation in binding affinities from − 7.6 to − 8.5 kcal/mol. In contrary, the binding affinities of the eliminated 34 phytochemicals varied from − 4.0 to − 7.5 kcal/mol. The lowest binding affinity was detected in Isopropyl butyrate (−4.0 kcal/mol) (Table 1 & Fig. 2). ADME/T analyses of the accepted 18 phytochemicals revealed three final lead candidates, such as Chrysin-7-O-glucuronide (−8.2 kcal/mol), Oroxindin (−8.1 kcal/mol) and Oroxin A (−8.0 kcal/mol) as depicted in Fig. 3.

**Table 1:** Phytochemicals of *O. indicum* used for first-step-virtual screening with their PubChem CID, 2D structures, molecular weight and binding affinities.

Phytochemicals	PubChem CID	Chemical formula	Molecular weight (g/mol)	Binding affinities (kcal/mol)	Two dimensional chemical structures	References
Anthraquinone	6780	C_14_H_8_O_2_	208.21	-6.3		[18]
Aloe-emodin	10207	C_15_H_10_O_5_	270.24	-6.8		[18]
Baicalein	5281605	C_15_H_10_O_5_	270.24	-7.6		[19]
Oroxin A	5320313	C_21_H_20_O_10_	432.4	-8.0		[19]
Oroxin B	10077207	C_27_H_30_O_15_	594.5	-9.3		[19]
Baicalin	64982	C_27_H_30_O_15_	446.4	-8.1		[19]
Chrysin	5281607	C_15_H_10_O_4_	254.24	-7.6		[19]
Chrysin-7-O-glucuronide	14135335	C_21_H_18_O_10_	430.4	-8.2		[19]
Oroxylin A	5320315	C_16_H_12_O_5_	284.26	-7.2		[19]
Scutellarin	185617	C_21_H_18_O_12_	462.4	-7.9		[19]
2-Methyl-6-(4-methylphenyl)hept-2-en-4-one	558221	C_15_H_20_O	216.32	-6.1		[19]
Methyl hexadecanoate	8181	C_17_H_34_O_2_	270.5	-4.4		[19]
Isopropyl butyrate	61184	C_7_H_14_O_2_	130.18	-4.0		[19]
Dihydrobaicalein	9816931	C_15_H_12_O_5_	272.25	-7.6		[19]
Ellagic acid	5281855	C_14_H_6_O_8_	302.19	-7.2		[19]
Dihydrooroxylin A	5316733	C_16_H_14_O_5_	286.28	-7.0		[19]
Hispidulin	5281628	C_16_H_12_O_6_	300.26	-7.3		[19]
Apigenin	5280443	C_15_H_10_O_5_	270.24	-7.8		[19]
Ficusal	10496641	C_18_H_18_O_6_	330.3	-6.5		[19]
Balanophonin	23252258	C_20_H_20_O_6_	356.4	-6.8		[19]
Salicylic acid	338	C_7_H_6_O_3_	138.12	-4.7		[19]
4-Hydroxybenzoic acid	135	C_7_H_6_O_3_	138.12	-5.0		[19]
3,4-Dihydroxybenzoic acid	72	C_7_H_6_O_4_	154.12	-5.4		[19]
Isovanillin	12127	C_8_H_8_O_3_	152.15	-4.7		[19]
Beta-Hydroxypropiovanillone	75142	C_10_H_12_O_4_	196.2	-5.2		[19]
5-Hydroxy-7-methoxy-2-phenylchroman-4-one	73201	C_16_H_14_O_4_	270.28	-6.8		[19]
Stigmast-7-en-3-ol	3080632	C_29_H_50_O	414.7	-8.2		[19]
Kaempferol	5280863	C_15_H_10_O_6_	286.24	-7.8		[19]
2-Isopropenyl-2,3-dihydronaphtho[2,3-*b*]furan-4,9-dione	364109	C_15_H_12_O_3_	240.25	-6.7		[19]
Lapachol	3884	C_15_H_14_O_3_	242.27	-6.4		[19]
Biochanin A	5280373	C_16_H_12_O_5_	284.26	-7.0		[19]
Beta-sitosterol	222284	C_29_H_50_O	414.7	-7.7		[19]
Oroxindin	3084961	C_22_H_20_O_11_	460.4	-8.1		[19]
Quercetin	5280343	C_15_H_10_O_7_	302.23	-7.8		[19]
Lupeol	259846	C_30_H_50_O	426.7	-8.3		[19]
Pinosylvin	5280457	C_14_H_12_O_2_	212.24	-6.6		[19]
Dihydropinosylvin	442700	C_14_H_14_O_2_	214.26	-6.2		[19]
Rengyol	363707	C_8_H_16_O_3_	160.21	-5.4		[19]
Zarzissine	6400641	C_5_H_5_N_5_	135.13	-5.3		[19]
Adenosine	60961	C_10_H_13_N_5_O_4_	267.24	-5.8		[19]
Sitogluside	5742590	C_35_H_60_O_6_	576.8	-8.5		[19]
Pinocembrin	68071	C_15_H_12_O_4_	256.25	-6.8		[19]
Echinulin	115252	C_29_H_39_N_3_O_2_	461.6	-7.5		[19]
Ursolic acid	64945	C_30_H_48_O_3_	456.7	-9.1		[19]
D-Galactose	6036	C_6_H_12_O_6_	180.16	-4.9		[18]
Prunetin	5281804	C_16_H_12_O_5_	284.26	-7.0		[18]
Baicalein 6-O-glucoside	5321896	C_21_H_20_O_10_	432.4	-8.4		[18]
Octanoic acid	379	C_8_H_16_O_2_	144.21	-4.1		[18]
Myristic acid	11005	C_14_H_28_O_2_	228.37	-4.4		[18]
Palmitic acid	985	C_16_H_32_O_2_	256.42	-4.9		[18]
Oleic acid	445639	C_18_H_34_O_2_	282.5	-4.9		[18]
Linoleic acid	5280450	C_18_H_32_O_2_	280.4	-5.2		[18]
Sunitinib (Control)	5329102	C_22_H_27_FN_4_O_2_	398.5	-7.6		[34]

### 3.2 Molecular interaction analysis

**Fig. 4:**
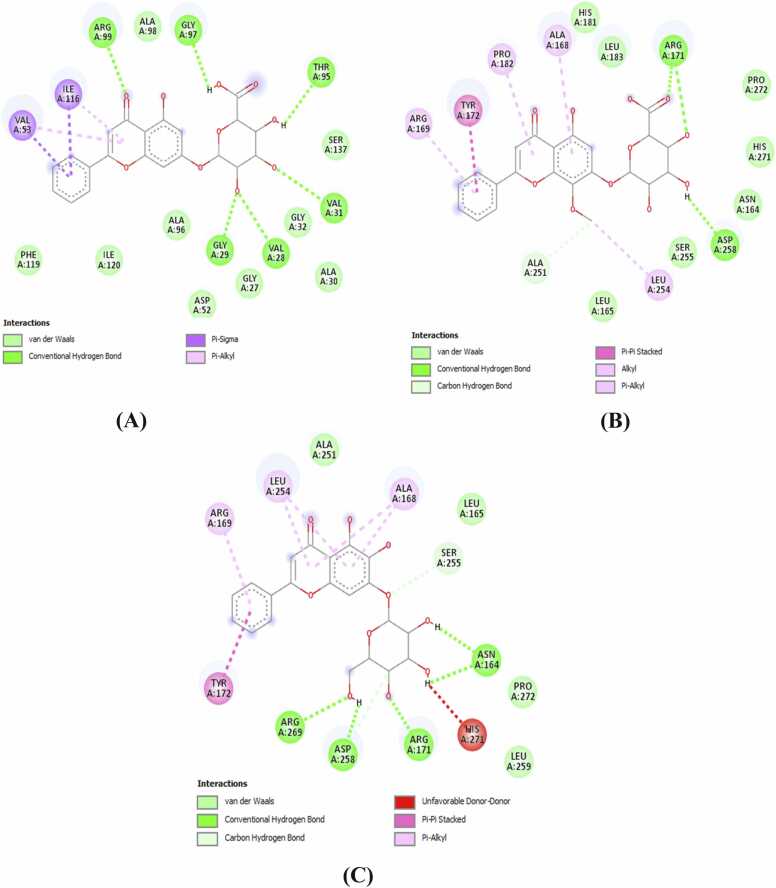
Two-dimensional molecular interactions of three lead compounds with amino acid residues of the macromolecular receptor: (A). Chrysin-7-O-glucuronide; (B). Oroxindin and (C). Oroxin A.

**Fig. 5:**
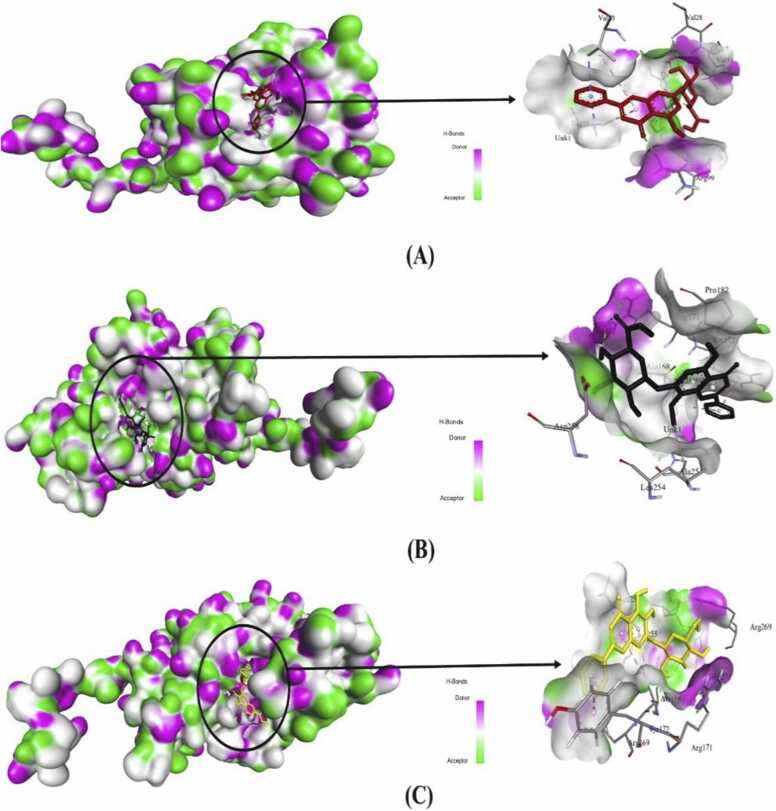
Docked complexes with three dimensional molecular interactions between the macromolecular receptor and top selected candidates to show the surface of hydrogen bond donating and accepting regions in the target protein LDHA: (A). Chrysin-7-O-glucuronide; (B). Oroxindin and (C). Oroxin A.

Molecular interactions between the phytoligands and the target protein were analyzed after visualization through BIOVIA Discovery Studio Visualizer that revealed noteworthy results. Both conventional hydrogen bonding and hydrophobic interactions were found in all the lead candidates (Fig. 4, Fig. 5).

Among the three top scoring candidates, Chrysin-7-O-glucuronide showed interactions with Val28, Gly29, Val31, Val53, Thr95, Gly97, Arg99 and Ile116 amino acid residues. This lead compound formed 6 conventional hydrogen bonds with Val28, Gly29, Val31, Thr95, Gly97 and Arg99 residues using bond distance of 3.04, 1.96, 3.06, 2.67, 2.07 and 2.61 Å, respectively. Only two residues, such as Val53 and Ile116 were associated in hydrophobic interactions with Chrysin-7-O-glucuronide. The second lead candidate Oroxindin interacted with Ala168, Arg169, Arg171, Tyr172, Pro182, Ala251, Leu254 and Asp258 amino acid residues where Arg171 formed two conventional hydrogen bonds with bond distances of 2.38 and 2.44 Å, respectively, and Asp258 formed one conventional hydrogen bond with a bond distance of 2.26 Å (Fig. 4, Fig. 5). Five residues, viz., Ala168, Arg169, Tyr172, Pro182 and Leu254 were involved in hydrophobic interactions. The third lead candidate, Oroxin A interacted with Asn164, Ala168, Arg169, Arg171, Tyr172, Leu254, Ser255, Asp258, Arg269 and His271 where four residues formed conventional hydrogen bonds. Asn164 formed two conventional hydrogen bonds with bond distances of 2.12 and 2.44 Å and the other three residues Arg171, Asp258 and Arg269 formed one conventional hydrogen bond each with a bond distance of 1.97, 2.61 and 2.69 Å, respectively. Hydrogen bond distance varied from 1.96 to 3.06 Å where the minimum (Gly29) and maximum (Val31) distances were observed with Chrysin-7-O-glucuronide (Table 2).

**Table 2:** Molecular interactions of Chrysin-7-O-glucuronide, Oroxindin, Oroxin A and Sunitinib with different amino acid residues of LDHA.

Ligands	Binding sites	Residues in conventional hydrogen bond formation (Distance in Å)	Hydrogen bond formed	Residues in hydrophobic interactions	Binding affinity (kcal/mol)
Chrysin-7-O-glucuronide	Val28, Gly29, Val31, Val53, Thr95, Gly97, Arg99, Ile116	Val28^(3.04)^, Gly29^(1.96)^, Val31^(3.06)^, Thr95^(2.67)^, Gly97^(2.07)^, Arg99^(2.61)^	6	Val53, Ile116	-8.2
Oroxindin	Ala168, Arg169, Arg171, Tyr172, Pro182, Ala251, Leu254, Asp258	Arg171^(2.38, 2.44)^, Asp258^(2.26)^	3	Ala168, Arg169, Tyr172, Pro182, Leu254	-8.1
Oroxin A	Asn164, Ala168, Arg169, Arg171, Tyr172, Leu254, Ser255, Asp258, Arg269, His271	Asn164^(2.12, 2.44)^, Arg171^(1.97)^, Asp258^(2.61)^, Arg269^(2.69)^	5	Ala168, Arg169, Tyr172, Leu254	-8.0
Sunitinib (Control)	Asn164, Leu165, Ala168, Arg169, Arg171, Tyr172, Pro182, Leu183, Ser237, Ala251, Leu254, Ser255, Asp258, His271	Ala168^(2.63)^	1	Leu165, Arg169, Tyr172, Pro182, Ala251	-7.6

### 3.3 Drug likeness analysis

Different pharmacodynamic and pharmacokinetic properties were evaluated via ADME/T analyses where all the top candidates revealed noteworthy results (Table 3). Oroxindin showed the highest molecular weight (460.39 g/mol) among the three candidates. Gastro-intestinal absorption property was found lower for all the lead compounds. None of the compounds revealed blood brain barrier permeability. Water solubility results were satisfactory as all the three candidates were found to be soluble in water. Drug likeness was estimated based on Lipinski’s Rules of Five and Ghose filter, where Chrysin-7-O-glucuronide showed zero violations in the Lipinski and Ghose criteria. Both Oroxindin and Oroxin A followed Ghose parameter with zero violations but showed one violation each in Lipinski’s Rules of Five which was acceptable. Cytochrome P450 enzyme inhibitory properties were also evaluated based on five isoforms, such as CYP1A2, CYP2C19, CYP2C9, CYP2D6 and CYP3A4 where all the three lead candidates showed no inhibition of these five isoforms.

**Table 3:** Evaluation of drug candidacy of Chrysin-7-O-glucuronide, Oroxindin and Oroxin A via ADME analysis.

Parameters		Chrysin-7-O-glucuronide	Oroxindin	Oroxin A
Physicochemical properties	Formula	C_21_H_18_O_10_	C_22_H_20_O_11_	C_21_H_20_O_10_
	Molecular weight	430.36 g/mol	460.39 g/mol	432.38 g/mol
	H-bond acceptor	10	11	10
	H-bond donors	5	5	6
	Molar refractivity	104.70	111.19	106.11
	TPSA	166.89 Å^2^	176.12 Å^2^	170.05 Å^2^
Lipophilicity	Log *P*_*o/w*_ (iLOGP)	2.05	1.30	2.54
	Log *P*_*o/w*_ (SILICOS-IT)	0.37	0.44	0.35
	Consensus Log *P*_*o/w*_	0.64	0.44	0.44
Pharmacokinetics	GI absorption	Low	Low	Low
	BBB permeant	No	No	No
	CYP1A2 inhibitor	No	No	No
	CYP2C19 inhibitor	No	No	No
	CYP2C9 inhibitor	No	No	No
	CYP2D6 inhibitor	No	No	No
	CYP3A4 inhibitor	No	No	No
	Log *K*_p_ (skin permeation)	-7.89 cm/s	-8.09 cm/s	-8.33 cm/s
Water Solubility	Log *S* (SILICOS-IT)	-2.81	-2.91	-2.69
	Solubility	6.62e-01 mg/ml; 1.54e-03 mol/l	5.65e-01 mg/ml; 1.23e-03 mol/l	8.77e-01 mg/ml; 2.03e-03 mol/l
	Class	Soluble	Soluble	Soluble
Drug likeness	Lipinski	Yes; 0 Violation	Yes; 1 Violation	Yes; 1 Violation
	Ghose	Yes	Yes	Yes
Medicinal Chemistry	Bioavailability Score	0.11	0.11	0.55
	PAINS	0 alert	0 alert	1 alert
	Synthetic accessibility	5.03	5.23	5.16

Pan-Assay Interference Compounds (PAINS) criterion revealed zero alerts for both Chrysin-7-O-glucuronide and Oroxindin and one alert for Oroxin A. Considering synthetic accessibility parameter, Chrysin-7-O-glucuronide scored better (5.03) than Oroxin A (5.16) and Oroxindin (5.23) (Table 3).

Toxicity properties were analyzed using two different servers for reliability of prediction where both the servers provided coherent satisfactory results (Table 4). ProToxII server revealed noteworthy results in the various parameters. Both Chrysin-7-O-glucuronide and Oroxindin revealed inactive status for all the criteria and subcriteria in ProToxII server. All the three lead compounds were predicted to have toxicity class 5 with an oral LD_50_ dose of 5000 mg/kg with 69.26% accuracy. Oroxin A also showed inactive status for all the parameters except one subcriterion, i.e*.*, Phosphoprotein p53 parameter of the Tox21-Stress response pathway where it indicated active status with 50% probability. StopTox server evaluated toxicity based on five toxicity endpoints, such as acute inhalation toxicity, acute oral toxicity, eye irritation and corrosion, skin sensitization and skin irritation and corrosion criteria where all the three lead candidates were found harmless and satisfactory (Table 4).

**Table 4:** Toxicity analysis for Chrysin-7-O-glucuronide, Oroxindin and Oroxin A.

Classification	Target		Prediction and probability		
			Chrysin-7-O-glucuronide	Oroxindin	Oroxin A
Organ toxicity	Hepatotoxicity		Inactive (0.73)	Inactive (0.79)	Inactive (0.82)
Toxicity end points	Carcinogenicity		Inactive (0.51)	Inactive (0.53)	Inactive (0.85)
	Immunotoxicity		Inactive (0.96)	Inactive (0.56)	Inactive (0.92)
	Mutagenicity		Inactive (0.74)	Inactive (0.61)	Inactive (0.76)
	Cytotoxicity		Inactive (0.81)	Inactive (0.91)	Inactive (0.69)
Tox21-Nuclear receptor signaling pathways	Aryl hydrocarbon Receptor (AhR)		Inactive (0.56)	Inactive (0.50)	Inactive (0.92)
	Androgen Receptor (AR)		Inactive (0.99)	Inactive (0.99)	Inactive (0.90)
	Androgen Receptor Ligand Binding Domain (AR-LBD)		Inactive (0.96)	Inactive (0.98)	Inactive (0.98)
	Aromatase		Inactive (0.94)	Inactive (0.93)	Inactive (1.0)
	Estrogen Receptor Alpha (ER)		Inactive (0.73)	Inactive (0.75)	Inactive (0.91)
	Estrogen Receptor Ligand Binding Domain (ER-LBD)		Inactive (0.83)	Inactive (0.79)	Inactive (0.99)
	Peroxisome Proliferator Activated Receptor Gamma (PPAR- γ)		Inactive (0.88)	Inactive (0.95)	Inactive (0.99)
Tox21-Stress response pathways	Nuclear factor (erythroid-derived 2)-like 2/antioxidant responsive element		Inactive (0.95)	Inactive (0.92)	Inactive (0.98)
	Heat shock factor response element (HSE)		Inactive (0.95)	Inactive (0.92)	Inactive (0.98)
	Mitochondrial Membrane Potential (MMP)		Inactive (0.61)	Inactive (0.52)	Inactive (0.98)
	Phosphoprotein (Tumor Suppressor) p53		Inactive (0.81)	Inactive (0.83)	**Active** (0.50)
	ATPase family AAA domain-containing protein 5 (ATAD5)		Inactive (0.83)	Inactive (0.71)	Inactive (1.0)
Acute inhalation toxicity			No	No	No
Acute oral toxicity			No	No	No
Eye irritation and corrosion			No	No	No
Skin sensitization			No	No	No
Skin irritation and corrosion			No	No	No

### 3.4 Molecular dynamics simulation

Molecular dynamics (MD) simulation exhibited small variation in RMSD, RMSF, Rg, SASA and intermolecular hydrogen bonds in the three lead compounds, such as, Chrysin-7-O-glucuronide, Oroxindin and Oroxin A. The MD trajectory was investigated for evaluating different systems employed in MD simulations and the findings are presented in Table 5.

**Table 5:** MD trajectory analysis using mean values for different systems.

No.	Systems	RMSD (nm)	RMSF (nm)	Rg (nm)	SASA (nm^2^)
1	Apoprotein	0.50	0.18	2.05	146.59
2	Sunitinib-protein complex	0.50	0.18	2.01	140.62
3	Chrysin-7-O-glucuronide-protein complex	0.64	0.19	2.02	147.37
4	Oroxindin-protein complex	0.70	0.17	1.99	144.16
5	Oroxin A-protein complex	0.61	0.17	2.01	141.97

**Fig. 6:**
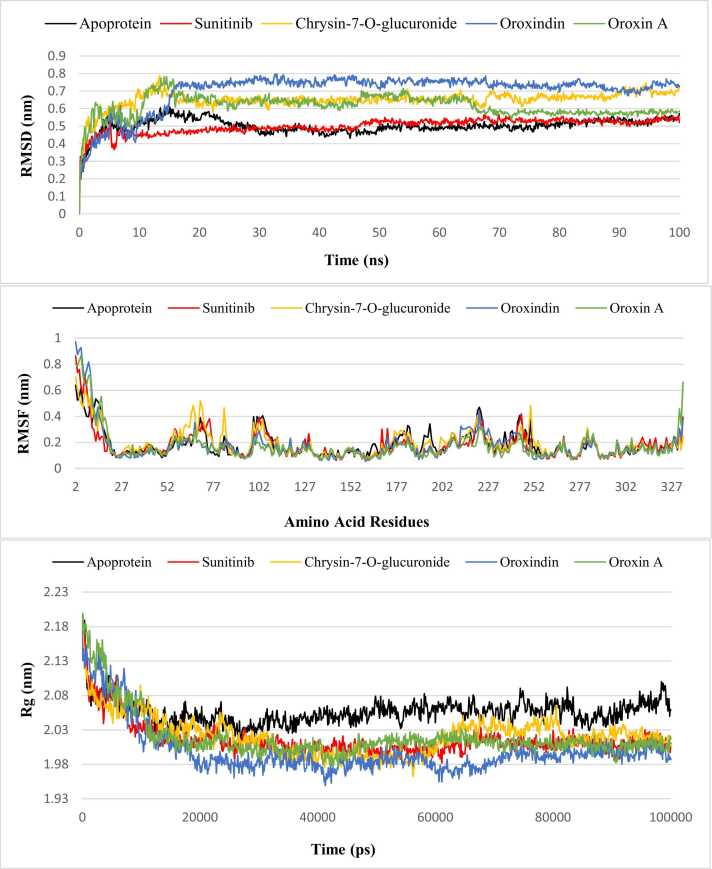
Molecular dynamics simulation for 100 ns depicting RMSD, RMSF and Rg properties of the lead candidates Chrysin-7-O-glucuronide, Oroxindin and Oroxin A along with Sunitinib and apoprotein.

RMSD, RMSF and Rg properties were evaluated for the whole 100 ns trajectory as shown in Fig. 6. RMSD graph revealed some initial fluctuations from 0 ns to 18 ns. After 18 ns, all the compounds showed good amount of stability up to 100 ns without any major fluctuations. Mean RMSD values were found the same (0.50 nm) in apoprotein and Sunitinib-protein complex. The other three systems involving the three lead compounds showed mean RMSD values of 0.64, 0.70 and 0.61 nm, for Chrysin-7-O-glucuronide, Oroxindin and Oroxin A complexes, respectively. Therefore, considering mean RMSD criterion, Oroxin A showed better stability among the three lead candidates (Table 5). After 68 ns, Oroxin A aligned closely with the Sunitinib-protein complex and the apoprotein.

Mean RMSF values were evaluated where both apoprotein and Sunitinib-protein complex showed same value (0.18 nm). The mean RMSF in Chrysin-7-O-glucuronide was found to be 0.19 nm which was slightly higher than those of apoprotein and the control-complex. The Oroxindin-protein complex and Oroxin A-protein complex showed the same mean RMSF value (0.17 nm). Therefore, both Oroxindin and Oroxin A complexes were found to have better regional flexibility profile than Chrysin-7-O-glucuronide, apoprotein and the Sunitinib-protein complex. The average distance of the lead compounds at binding pocket was analyzed that revealed a mean RMSF distance of 1.6 Å for Chrysin-7-O-glucuronide, 1.3 Å for Oroxindin and 1.0 Å for Oroxin A.

**Fig. 7:**
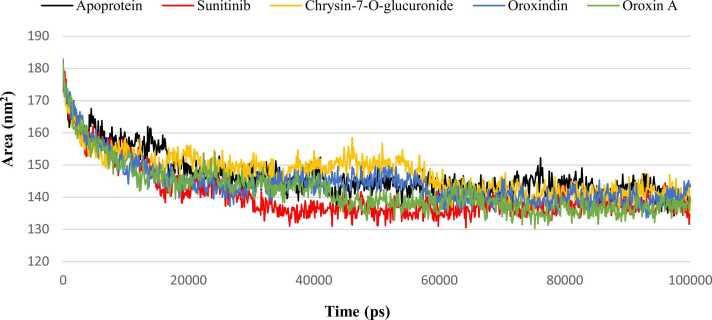
Molecular dynamics simulation for 100 ns depicting SASA values for the lead candidates Chrysin-7-O-glucuronide, Oroxindin and Oroxin A along with the control Sunitinib and apoprotein.

Rg graph demonstrated satisfactory results as compared to the apoprotein and control-complex. Mean Rg values were 2.05, 2.01, 2.02, 1.99 and 2.01 nm for apoprotein, control-complex, Chrysin-7-O-glucuronide-complex, Oroxindin-complex and Oroxin A-complex, respectively which indicated that Oroxindin-complex system showed the best structural compactness among all the five systems tested. Although fluctuations were observed for the top three lead complexes at various points of the trajectory, all of them interestingly converged to a single level thus representing close proximity with Sunitinib-complex near to 100 ns. SASA graph revealed compactness of the systems where the mean values of all the systems were almost similar with slight variations (Fig. 7). Among the lead candidates, Oroxin A-complex revealed the mean SASA value of 141.97 nm^2^ that showed a close proximity with the mean SASA value of Sunitinib-complex (140.62 nm^2^). Mean values of Chrysin-7-O-glucuronide-complex (147.37 nm^2^) and Oroxindin-complex (144.16 nm^2^) were found to be very close to the apoprotein (146.59 nm^2^).

**Fig. 8:**
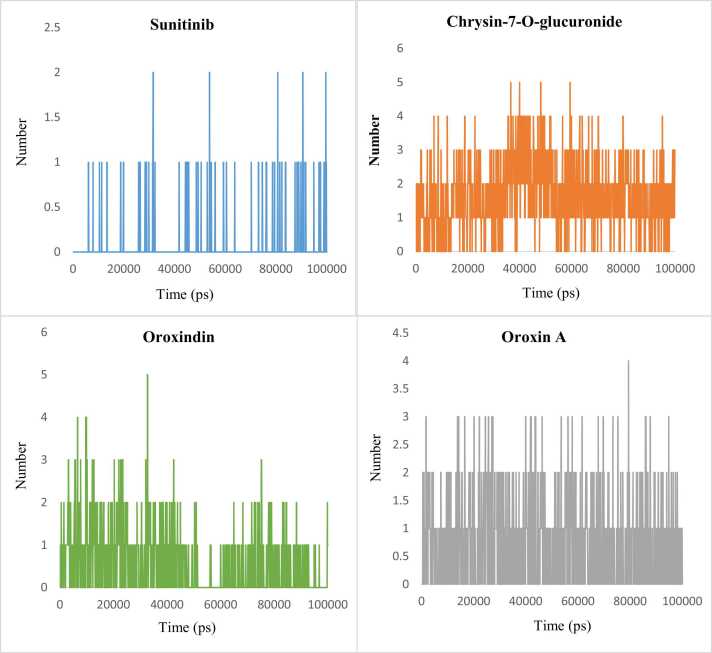
Number of hydrogen bonds formed between Lactate Dehydrogenase A and top three lead compounds along with the control Sunitinib.

All these findings indicated good structural compactness of the lead complexes. Number of hydrogen bonds between the LDHA and lead compounds revealed the highest average for Chrysin-7-O-glucuronide followed by Oroxin A and Oroxindin. All the lead candidates showed higher number of hydrogen bonds when compared to the control-complex during the 100 ns trajectory (Fig. 8).

### 3.5 Molecular mechanics/generalized born surface area (MM/GBSA) calculation

MM/GBSA free binding energy was calculated to eliminate false positive results and to corroborate the docking protocol. Prime module of Schrödinger revealed free binding energy values of − 45.72 kcal/mol, − 46.47 kcal/mol and − 37.25 kcal/mol for Chrysin-7-O-glucuronide-complex, Oroxindin-complex and Oroxin-A complex, respectively. All of the top selected complexes scored higher than the control Sunitinib-complex (−35.36) kcal/mol indicating that all the lead candidate-complexes were energetically more favorable than the control-complex (Table 6).

**Table 6:** Binding free energy estimation employing Prime MM/GBSA.

Complexes	MM/GBSA Δ G Bind (kcal/mol)	MM/GBSA Δ G Coulomb (kcal/mol)	MM/GBSA Δ G Covalent (kcal/mol)	MM/GBSA Δ G Hbond (kcal/mol)	MM/GBSA Δ G Lipo (kcal/mol)	MM/GBSA Δ G Bind Solve GB (kcal/mol)	MM/GBSA Δ G Bind vdW (kcal/mol)
Sunitinib-protein complex (control)	-35.56	-12.56	5.82	-0.27	-16.42	22.67	-33.63
Chrysin-7-O-glucuronide-protein complex	-45.72	-9.56	0.80	-0.71	-22.77	23.17	-36.65
Oroxindin-protein complex	-46.47	-23.76	3.74	-1.51	-16.35	20.371	-27.4
Oroxin A-protein complex	-37.25	-27.26	0.83	-2.74	-12.92	28.36	-23.53

### 3.6 Exploration of drug target class and similar structural analogs

**Fig. 9:**
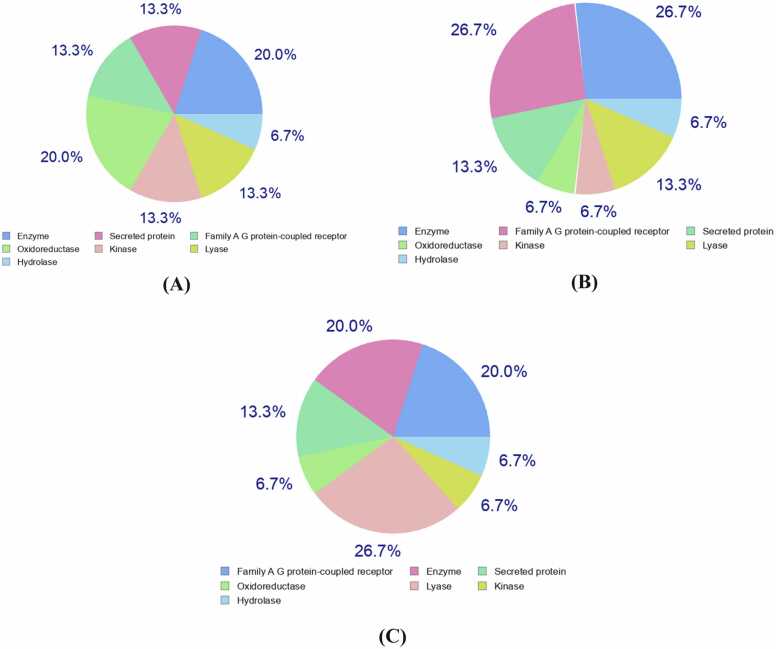
Drug target class predicted for Chrysin-7-O-glucuronide (A), Oroxindin (B) and Oroxin A (C).

**Fig. 10:**
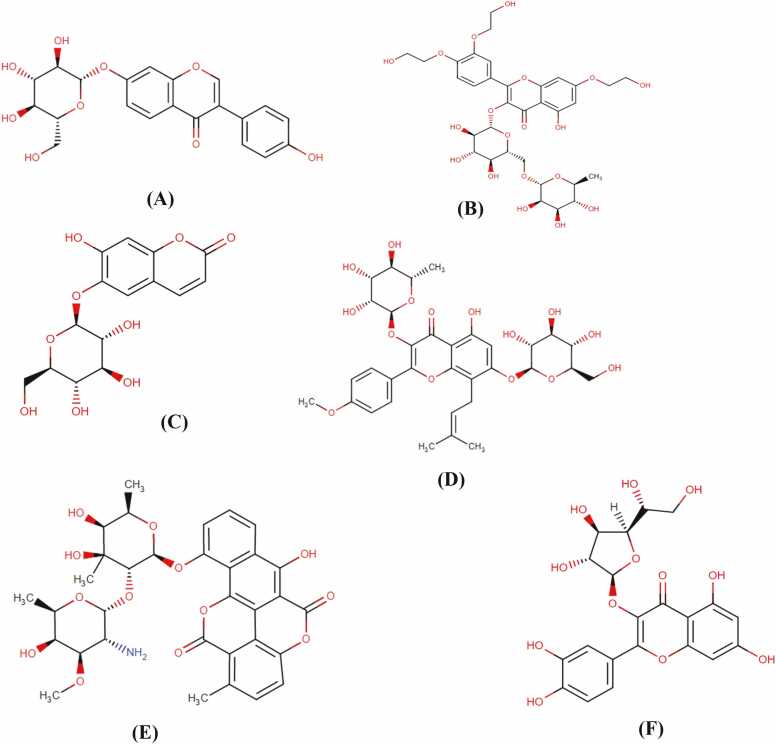
Two dimensional chemical structures of structurally similar analogs predicted for the top three phytochemicals: (A). Daidzin and (B). Troxerutin for Chrysin-7-O-glucuronide; (C). Esculin and (D). Icariin for Oroxindin; (E). Elsamitrucin and (F). Isoquercitrin for Oroxin A.

Aldolase reductase and Interleukin-2 were predicted as potential targets for Chrysin-7-O-glucuronide. Xanthine dehydrogenase was predicted as potential target for both Oroxindin and Oroxin A. In addition, Adenosine A1 receptor was found as potential target for Oroxindin and Aldolase reductase was revealed as promising target for Oroxin A. Most of the targets predicted for all the three leads belonged to enzymes (Table 7, Fig. 9). SwissSimilarity server predicted structurally similar analogs with good percentage of probability where Daidzin (68%) and Troxerutin (67%) were predicted to have similar structures of the lead compound Chrysin-7-O-glucuronide (Table 8, Fig. 10). Daidzin was found to be in the experimental stage and Troxerutin to be in the investigational stage. An FDA approved drug Esculin and another investigational drug Icariin were predicted to be analogous to Oroxindin with 55% and 62% probability, respectively. Elsamitrucin (59%) and Isoquercitrin (69%) were predicted as structurally similar analogs for Oroxin A, where both of the predicted drugs were found to be in the investigational stage.

**Table 7:** Target class prediction for Chrysin-7-O-glucuronide, Oroxindin and Oroxin A.

Ligands	Drug Targets	Common name	UniProt ID	Target Class
Chrysin-7-O-glucuronide	i. Aldolase reductase	AKR1B1	P15121	Enzyme
	ii. Interleukin-2	IL2	P60568	Secreted protein
Oroxindin	i. Adenosine A1 receptor	ADORA1	P30542	Family A G protein-coupled receptor
	ii. Xanthine dehydrogenase	XDH	P47989	Oxidoreductase
Oroxin A	i. Aldolase reductase	AKR1B1	P15121	Enzyme
	ii. Xanthine dehydrogenase	XDH	P47989	Oxidoreductase

**Table 8:** Structurally similar analogs for Chrysin-7-O-glucuronide, Oroxindin and Oroxin A.

Lead candidates	Structurally Similar Analogs	Drug Bank ID	Score	Status
Chrysin-7-O-glucuronide	i. Daidzin	DB02115	68%	Experimental
	ii. Troxerutin	DB13124	67%	Investigational
Oroxindin	i. Esculin	DB13155	55%	Approved
	ii. Icariin	DB12052	62%	Investigational
Oroxin A	i. Elsamitrucin	DB05129	59%	Investigational
	ii. Isoquercitrin	DB16403	69%	Investigational

## 4 Discussion

Computer aided structure-based drug design approach provides a rapid and cost-effective strategy to explore anticancer potential of various natural compounds to develop lead candidates targeting cancer [49], [50], [51]. In the current study, 52 phytochemicals from *O. indicum* were investigated utilizing a structure-based drug design approach to determine their anticancer potential targeting Lactate Dehydrogenase A enzyme *in silico.* Molecular docking analysis aided to screen out 65% of the total compounds immediately and further ADME/T analyses unveiled the top three lead candidates, such as Chrysin-7-O-glucuronide (−8.2 kcal/mol), Oroxindin (−8.1 kcal/mol) and Oroxin A (−8.0 kcal/mol). Molecular interaction displayed that both Oroxindin and Oroxin A interacted with Ala168, Arg171, Tyr172 and Asp258 residues which can be potential drug surface hotspots. Both hydrogen bonding and hydrophobic interactions were found in the lead candidates. Considering the number of conventional hydrogen bonds, all the three lead compounds showed much higher number than the control drug Sunitinib. Hydrogen bonds are crucial in regulating the specificity of ligand-macromolecule interactions [52]. Hydrophobic interactions between the lead candidates and receptor enhance the stability of docked complexes [53].

Chrysin-7-O-glucuronide is commonly present in *Scutellaria baicalensis* as a flavonoid [54]. This compound has been reported to exert inhibitory effects *in vitro* against alpha-amylase and alpha-glucosidase enzymes and thereby plays a vital role in treating diabetes [55]. Certain types of cancer cells express Breast Cancer Resistance Protein (BCRP), and its overexpression has been shown to be a significant contributor to the resistance of chemotherapeutic treatment [56]. Chrysin-7-O-glucuronide production considerably decreased when BCRP was silenced, showing that glucuronide is a substrate of BCRP, according to RNA-mediated silencing studies in HeLa cells [57]. While in an animal study, it was revealed that the impact of BCRP on Chrysin and its conjugates was limited as the PK parameters were not altered significantly in BCRP knockout mice compared to those in wild-type mice [58]. Kseibati et al. [59] reported that Chrysin administered orally at a dose of 50 mg/kg improved bleomycin-induced pulmonary fibrosis. Chrysin reduced hydroxyproline content, decreased TGF1 protein expression, lowered the activity of lactate dehydrogenase (LDH), and decreased lipid peroxidation. Chrysin-7-O-glucuronide, as a conjugate of Chrysin, may inhibit certain biotransformation enzymes (CYP2C9). Therefore, high intake of Chrysin-7-O-glucuronide might interrupt the transport and/or biotransformation of drugs [60]. In an *in vivo* rodent experiment peak plasma concentration of Chrysin-7-O-glucuronide was found considerably higher than that of Chrysin [61]. In mice, 20 mg/kg oral dose of Chrysin caused 160 nM peak plasma concentrations of Chrysin-7-O-glucuronide [58].

Oroxindin is a flavonoid which is commonly found in *O. indicum* and several other plants. This phytocompound has been reported in various *in vitro* studies to possess antimicrobial and anti-inflammatory properties [62], [63]. Oroxin A is another flavonoid usually isolated from *O. indicum* and it has been reported to possess significant inhibitory properties against breast cancer proliferation by generating significant endoplasmic reticulum stress and senescence [64].

Servers involving computational ADME and toxicity analyses have improved greatly in recent years with the incorporation of machine learning methods which have facilitated rapid analyses to evaluate various pharmacokinetic, pharmacodynamic and toxicity properties of drug-like compounds [65]. The present investigation revealed favorable ADME/T properties for Chrysin-7-O-glucuronide, Oroxindin and Oroxin A. In current drug discovery, interaction of lead compounds with cytochrome P450 (CYP) is considered as a crucial indicator. CYP isoforms exert regulatory effects in transformation, cellular metabolism, and excretion of drugs, and found to be critical in detoxification of foreign substances [66]. All lead candidates were found not to inhibit any of the CYP isoforms that was satisfactory. Lipinski’s rules of five was proposed when the drug discovery paradigm transitioned from phenotypic screening to combinatorial chemistry and high-throughput screening during mid- to late 1990’s [67]. The five principles constituted an investigation of compounds that passed Phase I and entered into the Phase II (clinical trials) by linking physicochemical characteristics, permeability, solubility, and oral bioavailability, thus acting as a crucial component in drug-likeness evaluation [68]. In the present study, Chrysin-7-O-glucuronide followed all the five rules of Lipinski with zero violations and the other two lead compounds followed the parameter with 1 violation each, which were acceptable. Pan Assay Interference Compounds (PAINS) criterion revealed zero alerts for both Chrysin-7-O-glucuronide and Oroxindin indicating that these leads would not cause false positive results [69]. Oroxin A only showed one violation in PAINS alert test, which was acceptable. All the investigated compounds revealed moderate synthetic accessibility where Chrysin-7-O-glucuronide scored better among the three compounds [70]. Toxicity properties revealed satisfactory results with no major side effects considering various toxicity endpoints and other parameters, and further corroborated our choice of the top three lead candidates. However, Computational ADME/T analyses encounter some limitations and are not completely error-free. Three essential elements make up a predictive ADME/T model: experimental data used to train the model; molecular structure descriptors that can be associated with the experimental data; and the right modeling technology. Lack of high-quality experimental data on which to develop models is one of the obstacles to improved prediction of organism-based features [71].

Molecular dynamics simulation unveiled significant structural stability and compactness for the top ligand-protein complexes compared to the control-complex and apoprotein LDHA. Higher values of RMSD, RMSF, Rg and SASA are indicative of higher degrees of flexibility and instability [66]. Rg and SASA provide insights regarding the global stability of the protein tertiary structures and ligands [72]. As mass-weighted RMSD for a group of atoms relative to their common mass center, Rg is often used to estimate whether a complex is stably folded or not [73]. Thus, the structure stability, within a valid MD simulation, is correlated to Rg values reaching a plateau around the average values [74]. In the current study, Oroxindin-complex showed better Rg values from 20 to 65 ns as compared to the control-complex and other two lead candidates, which is suggestive of significant stability, compactness and good accommodation within the protein pocket. After that with a very slight fluctuation, Oroxindin-complex became stable again at close to 80 ns up to 100 ns with lower Rg values than the control-complex. Oroxin A-complex followed the control-complex closely from 20 up to 100 ns indicating that Oroxin A holds the folding behavior of the protein. Chrysin-7-O-glucuronide-complex slightly fluctuated from 60 to 80 ns due to floppy packing but became stable again near 100 ns. With no major abrupt fluctuations after 20 ns, all the three lead candidate-complexes maintained the folding behavior of the protein Lactate Dehydrogenase A.

SASA measures the biomolecular surface area of the protein that is accessible to the solvent molecules. Reduced SASA values indicate relative structural shrinkage of protein-ligand complexes due to the influence of solvent surface charges, resulting in more compact and stable conformations [75]. The present investigation showed similar kind of SASA profiles for all the three lead candidate-complexes up to 40 ns that indicates similar kind of protein-ligand interactions. After 40 ns, SASA values decreased for Oroxin A-complex up to 50 ns and then became almost stable up to 100 ns showing close proximity with the control complex. This suggests the truncated nature of the complex across the simulation time that attributes to better stability. Oroxindin-complex showed reduced SASA value after 60 ns and closely aligned with the control-complex up to 100 ns for better compactness. After 40 ns, Chrysin-7-O-glucuronide-complex showed elevated SASA values close to 60 ns that might confer the migration of Chrysin-7-O-glucuronide towards the solvent side during this simulation time frame where the protein cavity became highly solvated and minimally compacted. After 60 ns, Chrysin-7-O-glucuronide-complex showed better stability up to 100 ns with no major fluctuations.

The present study showed close similarity among the lead complexes with lower values of the aforesaid criteria as compared to the control complex which strengthened Chrysin-7-O-glucuronide, Oroxindin and Oroxin A as lead candidates. MM/GBSA free binding energy was evaluated for all the three top ligand-protein complexes where Oroxindin-protein complex showed better results than other systems tested. This is considered as a revolutionary strategy involving quantum-mechanics/molecular-mechanics properties for estimating relative binding energies [76].

Drug target prediction aids in the discovery of new targets for the top selected candidates, whereas prediction of structurally similar analogs facilitates drug design process targeting the same receptor [77]. Various enzymes have been found as the target class in most of the cases for all the top three candidates which would be useful for conducting further studies. Daidzin and Troxerutin were predicted as similar analogs for Chrysin-7-O-glucuronide. Daidzin has been reported to show suppressive effects on rat prostate carcinogenesis as well as to inhibit growth and induce apoptosis in HeLa cell lines [78], [79]. Several *in vitro* investigations using various cancer cell lines demonstrated anticancer and cytotoxic properties of Troxerutin [80], [81], [82]. Esculin, an FDA approved drug was predicted to be analogous to Oroxindin which has been shown to have anticancer activities in glioblastoma, lung cancer, and breast cancer [83], [84], [85]. Similarly, Icariin, another analog of Oroxindin has been found to inhibit glioblastoma and gallbladder cancer [86], [87]. Elsamitrucin and Isoquercitrin were predicted as structurally similar analogs to Oroxin A, where both have been reported to have anticancer properties [88], [89].

Various computational studies have been attempted to explore novel inhibitors targeting LDHA [90], [91], [92]. Medicinal plants can unleash new avenues for discovering new effective inhibitors with insignificant side effects that can exert anti-cancer potential targeting LDHA. Although few bioinformatic approaches have been taken to explore the inhibitory potentials of *O. indicum* phytochemicals [93], [94], [95], no attempts have been undertaken to evaluate its anti-cancer potential *in silico* targeting Lactate Dyhydrogenase A. Therefore, the present study could unveil a new window on the discovery of novel LDHA inhibitors employing *Oroxylum indicum* phytochemicals against cancer.

## 5 Conclusion

The present investigation explored the anticancer efficacy of *Oroxylum indicum* using a variety of bioinformatic approaches to reveal novel drug candidates targeting Lactate Dehydrogenase A. Molecular docking unleashed 18 compounds initially from 52 phytoligands which were further processed through ADME and toxicity analyses. Finally, three potential candidates were identified, such as Chrysin-7-O-glucuronide, Oroxindin and Oroxin A wherein Chrysin-7-O-glucuronide showed the best binding affinity in molecular docking analysis. All the three lead compounds revealed favorable pharmacokinetic and pharmacodynamic properties with no major side effects. Furthermore, the top selected drug candidates exhibited noteworthy conformational stability and compactness in 100 ns molecular dynamics simulation. MM/GBSA study revealed Chrysin-7-O-glucuronide as the best lead candidate. Finally, we recommend further *in vivo* investigation for experimental validation of our findings.

## Funding

This research was not supported by any external funding.

## CRediT authorship contribution statement

**Sheikh Sunzid Ahmed**: Conceptualization, Methodology, Software, Data curation, Writing − original draft preparation. **M. Oliur Rahman**: Conceptualization, Methodology, Data curation, Visualization, Validation, Supervision, Writing − review & editing. **Ali S. Alqahtani**: Visualization, Validation, Writing − review & editing, Funding acquisition. **Nahid Sultana**: Investigation, Visualization, Writing − original draft preparation. **Omer M. Almarfadi**: Methodology, Software, Visualization. **M. Ajmal Ali**: Visualization, Writing − review & editing. **Joongku Lee**: Visualization, Writing − review & editing.

## Declaration of Competing Interest

The authors declare that they have no known competing financial interests or personal relationships that could have appeared to influence the work reported in this paper.

## Data Availability

No data was used for the research described in the article.

## References

[1] Zhang W., Wang C., Hu X., Lian Y., Ding C., Ming L. (2022). Inhibition of LDHA suppresses cell proliferation and increases mitochondrial apoptosis via the JNK signaling pathway in cervical cancer cells. Oncol. Rep..

[2] Liberti M.V., Locasale J.W. (2016). The Warburg effect: How does it benefit cancer cells?. Trends Biochem. Sci..

[3] Cascardo F., Anselmino N., Páez A., Labanca E., Sanchis P., Antico-Arciuch V., Navone N., Gueron G., Vázquez E., Cotignola J. (2021). HO-1 modulates aerobic glycolysis through LDH in prostate cancer cells. Antioxidants.

[4] Feng Y., Xiong Y., Qiao T., Li X., Jia L., Han Y. (2018). Lactate dehydrogenase A: A key player in carcinogenesis and potential target in cancer therapy. Cancer Med.

[5] Tayel F., Mahfouz M.E., Salama A.F., Mansour M.A. (2021). Ethoxyquin inhibits the progression of Murine Ehrlich Ascites Carcinoma through the inhibition of autophagy and LDH. Biomedicines.

[6] Dhal A.K., Pani A., Mahapatra R.K., Yun S.I. (2018). *In-silico* screening of small molecule inhibitors against Lactate Dehydrogenase (LDH) of *Cryptosporidium parvum*. Comput. Biol. Chem..

[7] Sun R., Li X., Li Y., Zhang X., Li X., Li X., Shi Z., Bao J. (2015). Screening of novel inhibitors targeting lactate dehydrogenase A via four molecular docking strategies and dynamics simulations. J. Mol. Model..

[8] Giatromanolaki A., Sivridis E., Gatter K.C., Turley H., Harris A.L., Koukourakis M.I. (2006). Lactate dehydrogenase 5 (LDH-5) expression in endometrial cancer relates to the activated VEGF/VEGFR2 (KDR) pathway and prognosis. Gynecol. Oncol..

[9] Wang Z.Y., Loo T.Y., Shen J.G., Wang N., Wang D.M., Yang D.P., Mo S.L., Guan X.Y., Chen J.P. (2012). LDH-A silencing suppresses breast cancer tumorigenicity through induction of oxidative stress mediated mitochondrial pathway apoptosis. Breast Cancer Res. Treat..

[10] Rong Y., Wu W., Ni X., Kuang T., Jin D., Wang D., Lou W. (2013). Lactate dehydrogenase A is overexpressed in pancreatic cancer and promotes the growth of pancreatic cancer cells. Tumor Biol..

[11] Yao F., Zhao T., Zhong C., Zhu J., Zhao H. (2013). LDHA is necessary for the tumorigenicity of esophageal squamous cell carcinoma. Tumor Biol..

[12] Miao P., Sheng S., Sun X., Liu J., Huang G. (2013). Lactate dehydrogenase A in cancer: A promising target for diagnosis and therapy. IUBMB Life.

[13] Friberg A., Rehwinkel H., Nguyen D., Pütter V., Quanz M., Weiske J., Eberspächer U., Heisler I., Langer G. (2020). Structural evidence for isoform-selective allosteric inhibition of lactate dehydrogenase A. ACS Omega.

[14] Cheng C.S., Tan H.Y., Wang N., Chen L., Meng Z., Chen Z., Feng Y. (2021). Functional inhibition of lactate dehydrogenase suppresses pancreatic adenocarcinoma progression. Clin. Transl. Med..

[15] Franczak M., Kutryb-Zajac B., El Hassouni B., Giovannetti E., Granchi C., Minutolo F., Smolenski R.T., Peters G.J. (2022). The effect of lactate dehydrogenase-A inhibition on intracellular nucleotides and mitochondrial respiration in pancreatic cancer cells. Nucl Nucl Nucl.

[16] Zhong M., Fang Z., Ruan B., Xiong J., Li J., Song Z. (2022). LINC01128 facilitates the progression of pancreatic cancer through up-regulation of LDHA by targeting miR-561-5p. Cancer Cell Int.

[17] N. Sultana, Taxonomy, Propagation and chemical properties of selected anticancerous plants of Bangladesh, 2017, Ph.D. Thesis submitted to the University of Dhaka (unpublished).

[18] Singh V., Chaudhary A.K. (2011). A review on the taxonomy, ethnobotany, chemistry and pharmacology of *Oroxylum indicum* Vent. Indian J. Pharm. Sci..

[19] Jagetia G.C. (2021). A review on the medicinal and pharmacological properties of traditional ethnomedicinal plant Sonapatha, Oroxylum indicum. Sinusitis.

[20] Adelusi T.I., Oyedele A.Q.K., Boyenle I.D., Ogunlana A.T., Adeyemi R.O., Ukachi C.D., Idris M.O., Olaoba O.T., Adedotun I.O., Kolawole O.E., Xiaoxing Y., Abdul-Hammed M. (2022). Molecular modeling in drug discovery. Inform. Med. Unlocked.

[21] Fan J., Fu A., Zhang L. (2019). Progress in molecular docking. Quant. Biol..

[22] Dias R., de Azevedo J., Walter F. (2008). Molecular docking algorithms. Curr. Drug Targets.

[23] Meng X.Y., Zhang H.X., Mezei M., Cui M. (2011). Molecular docking: A powerful approach for structure-based drug discovery. Curr. Comput. Aided Drug Des..

[24] Brooijmans N., Kuntz I.D. (2003). Molecular recognition and docking algorithms. Annu. Rev. Biophys. Biomol. Struct..

[25] Schentag J.J., Gilliland K.K., Paladino J.A. (2001). What have we learned from pharmacokinetic and pharmacodynamic theories?. Clin. Infect. Dis..

[26] Kar S., Leszczynski J. (2020). Open access *in silico* tools to predict the ADMET profiling of drug candidates. Expert Opin. Drug Disco.

[27] Moroy G., Martiny V.Y., Vayer P., Villoutreix B.O., Miteva M.A. (2012). Toward *in silico* structure-based ADMET prediction in drug discovery. Drug Discov. Today.

[28] Kumar A., Kini S.G., Rathi E. (2021). A recent appraisal of artificial intelligence and *in silico* ADMET prediction in the early stages of drug discovery. Mini-Rev. Med. Chem..

[29] Berman H.M., Westbrook J., Feng Z., Gilliland G., Bhat T.N., Weissig H., Shindyalov I.N., Bourne P.E. (2000). The protein data bank. Nucleic Acids Res.

[30] Kolappan S., Shen D.L., Mosi R., Sun J., McEachern E.J., Vocadlo D.J., Craig L. (2015). Structures of lactate dehydrogenase A (LDHA) in apo, ternary and inhibitor-bound forms. Acta Crystallogr Sect. D: Biol. Crystallogr.

[31] Morris G.M., Huey R., Olson A.J. (2008). Using autodock for ligand‐receptor docking. Curr. Protoc. Bioinform..

[32] Guex N., Peitsch M.C. (1997). SWISS‐MODEL and the Swiss‐Pdb Viewer: An environment for comparative protein modeling. Electrophoresis.

[33] O'Boyle N.M., Banck M., James C.A., Morley C., Vandermeersch T., Hutchison G.R. (2011). Open Babel: An open chemical toolbox. J. Chemoinformatics.

[34] Broggini T., Stange L., Lucia K.E., Vajkoczy P., Czabanka M. (2022). Endothelial EphrinB2 regulates Sunitinib therapy response in Murine Glioma. Life.

[35] Talluri S. (2021). Molecular docking and virtual screening based prediction of drugs for COVID-19. Comb. Chem. High. Throughput Screen..

[36] Halgren T.A. (1996). Merck molecular force field. I. Basis, form, scope, parameterization, and performance of MMFF94. J. Comput. Chem..

[37] Trott O., Olson A.J. (2010). AutoDock Vina: Improving the speed and accuracy of docking with a new scoring function, efficient optimization, and multithreading. J. Comput. Chem..

[38] Daina A., Michielin O., Zoete V. (2017). SwissADME: A free web tool to evaluate pharmacokinetics, drug-likeness and medicinal chemistry friendliness of small molecules. Sci. Rep..

[39] Banerjee P., Eckert A.O., Schrey A.K., Preissner R. (2018). ProTox-II: A webserver for the prediction of toxicity of chemicals. Nucleic Acids Res.

[40] Borba J.V., Alves V.M., Braga R.C., Korn D.R., Overdahl K., Silva A.C., Hall S.U.S., Overdahl E., Kleinstreuer N., Strickland J., Allen D., Andrade C.H., Muratov E.N., Tropsha A. (2022). STopTox: An in silico alternative to animal testing for acute systemic and topical toxicity. Environ. Health Perspect..

[41] Abraham M.J., Murtola T., Schulz R., Páll S., Smith J.C., Hess B., Lindahl E. (2015). GROMACS: High performance molecular simulations through multi-level parallelism from laptops to supercomputers. SoftwareX.

[42] Schüttelkopf A.W., Van D.M. (2004). Aalten, PRODRG: a tool for high-throughput crystallography of protein–ligand complexes. Acta Crystallogr. Sect. D: Biol. Crystallogr..

[43] Meza J.C. (2010). Steepest descent. Wiley Interdiscip. Rev. Comput. Stat..

[44] Van Gunsteren W.F., Berendsen H.J. (1988). A leap-frog algorithm for stochastic dynamics. Mol. Simul..

[45] Ongaro A., Oselladore E., Memo M., Ribaudo G., Gianoncelli A. (2021). Insight into the LFA-1/SARS-CoV-2 Orf7a complex by protein–protein docking, molecular dynamics, and MM-GBSA calculations. J. Chem. Inf. Model.

[46] Kushwaha P.P., Maurya S.K., Singh A., Prajapati K.S., Singh A.K., Shuaib M., Kumar S. (2021). *Bulbine frutescens* phytochemicals as novel ABC-transporter inhibitor: A molecular docking and molecular dynamics simulation study. J. Cancer Metastas-.-. Treat..

[47] Daina A., Michielin O., Zoete V. (2019). SwissTargetPrediction: Updated data and new features for efficient prediction of protein targets of small molecules. Nucleic Acids Res.

[48] Zoete V., Daina A., Bovigny C., Michielin O. (2016). SwissSimilarity: A web tool for low to ultra high throughput ligand-based virtual screening. J. Chem. Inf. Model.

[49] Carabet L.A., Rennie P.S., Cherkasov A. (2018). Therapeutic inhibition of Myc in cancer. Structural bases and computer-aided drug discovery approaches. Int. J. Mol. Sci..

[50] Mendie L.E., Hemalatha S. (2022). Molecular docking of phytochemicals targeting GFRs as therapeutic sites for cancer: An in silico study. Appl. Biochem. Biotechnol..

[51] Patrício R.P., Videira P.A., Pereira F. (2022). A computer-aided drug design approach to discover tumour suppressor p53 protein activators for colorectal cancer therapy. Bioorg. Med. Chem..

[52] Gancia E., Montana J.G., Manallack D.T. (2001). Theoretical hydrogen bonding parameters for drug design. J. Mol. Graph.

[53] van Dijk E., Hoogeveen A., Abeln S. (2015). The hydrophobic temperature dependence of amino acids directly calculated from protein structures. PLoS Comput. Biol..

[54] Horvath C.R., Martos P.A., Saxena P.K. (2005). Identification and quantification of eight flavones in root and shoot tissues of the medicinal plant Huang-qin (*Scutellaria baicalensis* Georgi) using high-performance liquid chromatography with diode array and mass spectrometric detection. J. Chromatogr. A.

[55] Li K., Yao F., Xue Q., Fan H., Yang L., Li X., Sun L., Liu Y. (2018). Inhibitory effects against α-glucosidase and α-amylase of the flavonoids-rich extract from *Scutellaria baicalensis* shoots and interpretation of structure–activity relationship of its eight flavonoids by a refined assign-score method. Chem. Cent. J..

[56] Mao Q., Unadkat J.D. (2015). Role of the breast cancer resistance protein (BCRP/ABCG2) in drug transport - An update. AAPS J..

[57] Quan E., Wang H., Dong D., Zhang X., Wu B. (2015). Characterization of Chrysin glucuronidation in UGT1A1-overexpressing HeLa cells: Elucidating the transporters responsible for efflux of glucuronide. Drug Metab. Dispos..

[58] Ge S., Gao S., Yin T., Hu M. (2015). Determination of pharmacokinetics of Chrysin and its conjugates in wild-type FVB and BCRP1 knockout mice using a validated LC-MS/MS method. J. Agric. Food Chem..

[59] Kseibati M.O., Sharawy M.H., Salem H.A. (2020). Chrysin mitigates bleomycin-induced pulmonary fibrosis in rats through regulating inflammation, oxidative stress, and hypoxia. Int. Immunopharmacol..

[60] Mohos V., Fliszár-Nyúl E., Ungvári O., Bakos É., Kuffa K., Bencsik T., Zsidó B.Z., Hetényi C., Telbisz Á., Özvegy-Laczka C., Poór M. (2020). Effects of Chrysin and its major conjugated metabolites Chrysin-7-sulfate and Chrysin-7-glucuronide on Cytochrome P450 enzymes and on OATP, P-gp, BCRP, and MRP2 transporters. Drug Metab. Dispos..

[61] Noh K., Nepal M.R., Jeong K.S., Choi Y., Kang M.J., Kang W., Jeong H.G., Jeong T.C. (2016). Pharmacokinetic interaction of Chrysin with Caffeine in rats. Biomol. Ther..

[62] Chaudhuri P.K., Srivastava R., Kumar S., Kumar S. (2004). Phytotoxic and antimicrobial constituents of *Bacopa monnieri* and *Holmskioldia sanguinea*. Phytother. Res..

[63] Liu Q., Zuo R., Wang K., Nong F.F., Fu Y.J., Huang S.W., Pan Z.F., Zhang Y., Luo X., Deng X.L., Zhang X.X., Zhou L., Chen Y. (2020). Oroxindin inhibits macrophage NLRP3 inflammasome activation in DSS-induced ulcerative colitis in mice via suppressing TXNIP-dependent NF-κB pathway. Acta Pharmacol. Sin..

[64] He J., Du L., Bao M., Zhang B., Qian H., Zhou Q., Cao Z. (2016). Oroxin A inhibits breast cancer cell growth by inducing robust endoplasmic reticulum stress and senescence. Anti-Cancer Drugs.

[65] Tao L., Zhang P., Qin C., Chen S.Y., Zhang C., Chen Z., Zhu F., Yang S.Y., Wei Y.Q., Chen Y.Z. (2015). Recent progresses in the exploration of machine learning methods as in-silico ADME prediction tools. Adv. Drug Deliv. Rev..

[66] Ali M.C., Nur A.J., Khatun M.S., Dash R., Rahman M.M., Karim M.M. (2020). Identification of potential SARS-CoV-2 main protease inhibitors from Ficus carica Latex: An in-silico approach. J. Adv. Biotechnol. Exp. Ther..

[67] Pollastri M.P. (2010). Overview on the Rule of Five. Curr. Protoc. Pharmacol..

[68] Lipinski C.A. (2004). Lead-and drug-like compounds: The rule-of-five revolution. Drug. Discov. Today Technol..

[69] Baell J.B., Nissink J.W.M. (2018). Seven Year Itch: Pan-Assay Interference Compounds (PAINS) in 2017- Utility and Limitations. ACS Chem. Biol..

[70] Rahman A., Naheed N.H., Raka S.C., Qais N., Momen A.Z.M. (2020). Ligand-based virtual screening, consensus molecular docking, multi-target analysis and comprehensive ADMET profiling and MD stimulation to find out noteworthy tyrosine kinase inhibitor with better efficacy and accuracy. Adv. Trad. Med..

[71] Cheng F., Li W., Liu G., Tang Y. (2013). In Silico ADMET prediction: Recent advances, current challenges and future trends. Curr. Top. Med. Chem..

[72] Zaki A.A., Ashour A., Elhady S.S., Darwish K.M., Al-Karmalawy A.A. (2022). Calendulaglycoside A showing potential activity against SARS-CoV-2 main protease: Molecular docking, molecular dynamics, and SAR studies. J. Trad. Complement. Med..

[73] Joshi T., Joshi T., Sharma P., Chandra S., Pande V. (2021). Molecular docking and molecular dynamics simulation approach to screen natural compounds for inhibition of *Xanthomonas oryzae* pv. *Oryzae* by targeting peptide deformylase. J. Biomol. Struct. Dyn..

[74] Likić V.A., Gooley P.R., Speed T.P., Strehler E.E. (2005). A statistical approach to the interpretation of molecular dynamics simulations of calmodulin equilibrium dynamics. Protein Sci..

[75] Khan J., Sakib S.A., Mahmud S., Khan Z., Islam M.N., Sakib M.A., Emran T.B., Simal-Gandara J. (2021). Identification of potential phytochemicals from *Citrus limon* against main protease of SARS-CoV-2: Molecular docking, molecular dynamic simulations and quantum computations. J. Biomol. Struct. Dyn..

[76] Choudhary M.I., Shaikh M., tul-Wahab A., ur-Rahman A. (2020). In silico identification of potential inhibitors of key SARS-CoV-2 3CL hydrolase (Mpro) via molecular docking, MMGBSA predictive binding energy calculations, and molecular dynamics simulation. PLoS One.

[77] Ahmed S.R., Banik A., Anni S.M., Chowdhury M.M.H. (2021). Inhibitory potential of plant-derived metabolites against Zika virus: A computational-aided approach. Phytomed.

[78] Kato K., Takahashi S., Cui L., Toda T., Suzuki S., Futakuchi M., Sugiura S., Shirai T. (2000). Suppressive effects of dietary genistin and daidzin on rat prostate carcinogenesis. Jpn. J. Cancer Res.

[79] Yao Z., Xu X., Huang Y. (2021). Daidzin inhibits growth and induces apoptosis through the JAK2/STAT3 in human cervical cancer HeLa cells. Saudi J. Biol. Sci..

[80] Panat N.A., Singh B.G., Maurya D.K., Sandur S.K., Ghaskadbi S.S. (2016). Troxerutin, a natural flavonoid binds to DNA minor groove and enhances cancer cell killing in response to radiation, Chem.-Biol. Interact.

[81] Hassanshahi J., Mirzahosseini-Pourranjbar A., Hajializadeh Z., Kaeidi A. (2020). Anticancer and cytotoxic effects of troxerutin on HeLa cell line: An in-vitro model of cervical cancer. Mol. Biol. Rep..

[82] Yu J., Huang X., Cao M., Qian L., Shao L., Chinnathambi A., Alharbi S.A., Jian J. (2021). Anticancer effect of Troxerutin in human Non-Small-Cell lung cancer cell A549 and inhibition of tumor formation in BALB/c nude mice. J. Environ. Pathol. Toxicol..

[83] Mokdad-Bzeouich I., Kovacic H., Ghedira K., Chebil L., Ghoul M., Chekir-Ghedira L., Luis J. (2016). Esculin and its oligomer fractions inhibit adhesion and migration of U87 glioblastoma cells and in vitro angiogenesis. Tumor Biol..

[84] Mo M., Chen M.J., Huang Y., Jiang W., Qin Q.H., Liang Z.J., Yang W.P., Wei C.Y. (2020). Esculin inhibits proliferation of triple negative breast cancer cells by down-regulating FBI-1, Chin. J. Oncol..

[85] Goda M.S., Nafie M.S., Awad B.M., Abdel-Kader M.S., Ibrahim A.K., Badr J.M., Eltamany E.E. (2021). In vitro and in vivo studies of anti-lung cancer activity of *Artemesia judaica* L. crude extract combined with LC-MS/MS metabolic profiling, docking simulation and HPLC-DAD quantification. Antioxidants.

[86] Zhang D.C., Liu J.L., Ding Y.B., Xia J.G., Chen G.Y. (2013). Icariin potentiates the antitumor activity of gemcitabine in gallbladder cancer by suppressing NF-κB. Acta Pharmacol. Sin..

[87] Yang L., Wang Y., Guo H., Guo M. (2015). Synergistic anti-cancer effects of icariin and temozolomide in glioblastoma. Cell Biochem. Biophys..

[88] Goss G., Letendre F., Stewart D., Shepherd F., Schacter L., Hoogendoorn P., Eisenhauer E. (1994). Phase II study of elsamitrucin in non-small cell lung cancer. Invest. N. Drugs.

[89] Huang G., Tang B., Tang K., Dong X., Deng J., Liao L., Liao Z., Yang H., He S. (2014). Isoquercitrin inhibits the progression of liver cancer in vivo and in vitro via the MAPK signalling pathway. Oncol. Rep..

[90] Fang A., Zhang Q., Fan H., Zhou Y., Yao Y., Zhang Y., Huang X. (2017). Discovery of human Lactate Dehydrogenase A (LDHA) inhibitors as anticancer agents to inhibit the proliferation of MG-63 osteosarcoma cells. MedChemComm.

[91] Amr A.E.G.E., Mageid R.E.A., El-Naggar M., Naglah A.M., Nossier E.S., Elsayed E.A. (2020). Chiral Pyridine-3, 5-bis-(L-phenylalaninyl-L-leucinyl) Schiff Base peptides as potential anticancer agents: Design, synthesis, and molecular docking studies targeting Lactate Dehydrogenase-A. Molecules.

[92] Cheyad M.S., Al-qaisi A.H.J., Ahmed A. (2022). Synthesis, molecular docking and molecular dynamics simulation of 1, 4-bis (4, 5-diphenyl-1H-imidazol-2-yl) benzene as a potential inhibitor against LDHA. Appl. Nanosci..

[93] Poonacha S.K., Harishkumar M., Radha M., Varadarajan R., Nalilu S.K., Shetty S.S., Shetty P.K., Chandrashekharappa R.B., Sreenivas M.W., Bavabeedu S.K.B. (2021). Insight into OroxylinA-7-O-β-d-Glucuronide-enriched *Oroxylum indic*um bark extract in oral cancer HSC-3 cell apoptotic mechanism: Role of mitochondrial microenvironment. Molecules.

[94] Parvin M., Rahaman A., Sarkar A., Debnath S., De U.C., Mandal D.P., Bhattacharjee S. (2022). *Oroxylum indicum* stem bark extract reduces tumor progression by inhibiting the EGFR-PI3K-AKT pathway in an in vivo 4NQO-induced oral cancer model. J. Am. Coll. Nutr..

[95] Sultana A., Spriha S.E., Rahman S.A. (2022). Antioxidant, analgesic, antimicrobial and molecular docking studies of the leaves of *Oroxylum indicum* (L.) Kurz.. Dhaka Univ. J. Pharm. Sci..

