# Supplementary material for: Anticancer potential of phytochemicals from Oroxylum indicum targeting Lactate Dehydrogenase A through bioinformatic approach
Source: Toxicol Rep. 2022 Dec 14;10:56–75. doi: 10.1016/j.toxrep.2022.12.007 (PMC9792705; doi:10.1016/j.toxrep.2022.12.007)
Supplement: Supplementary file 1 — Supplementary material. [file mmc1.pdf]

Supplementary File 1

For materials and methods section 2.1 - Conversion from PDB to PDBQT command:

obabel –ipdb *.pdb –opdbqt –O*.pdbqt

For materials and methods section 2.2 - Energy minimization command:

obminimize –ff MMFF94 –n 2000 *.sdf

For materials and methods section 2.2 - Conversion from SDF to PDBQT command:

obabel –isdf *.sdf –opdbqt –O*.pdbqt
