# Supplementary material for: Anticancer potential of phytochemicals from Oroxylum indicum targeting Lactate Dehydrogenase A through bioinformatic approach
Source: Toxicol Rep. 2022 Dec 14;10:56–75. doi: 10.1016/j.toxrep.2022.12.007 (PMC9792705; doi:10.1016/j.toxrep.2022.12.007)
Supplement: Supplementary file 2 — Supplementary material. [file mmc2.pdf]

Supplementary File 2

Vina_windows.pl script:

```
#!/usr/bin/perl
print"Ligand_file:\t";
$ligfile=<STDIN>;
chomp $ligfile;
open (FH,$ligfile) || die "cannot open file\n";
@arr_file=<FH>;

for ($i=0;$i<@arr_file;$i++)
{

print"@arr_file[$i]\n";
@name=split(/\./,@arr_file[$i]);
}

for ($i=0;$i<@arr_file;$i++)
{
	chomp @arr_file[$i];
	print"@arr_file[$i]\n";
	system("Vina.exe --config conf_vs.txt --ligand @arr_file[$i] --log @arr_file[$i]_log.log");
}
```
